# The Role of 2D and 3D Echo in Mitral Stenosis

**DOI:** 10.3390/jcdd8120171

**Published:** 2021-12-03

**Authors:** Juan Manuel Monteagudo Ruiz, José Luis Zamorano Gómez

**Affiliations:** Cardiology Department, University Hospital Ramón y Cajal, 28034 Madrid, Spain; j5469m@gmail.com

**Keywords:** mitral stenosis, echocardiography, 3D echocardiography, mitral annulus calcification

## Abstract

Mitral stenosis is an important cause of heart valve disease globally. Echocardiography is the main imaging modality used to diagnose and assess the severity and hemodynamic consequences of mitral stenosis as well as valve morphology. Transthoracic echocardiography (TTE) is sufficient for the management of most patients. The focus of this review is the role of current two-dimensional (2D) and three-dimensional (3D) echocardiographic imaging for the evaluation of mitral stenosis.

## 1. Introduction

Mitral stenosis is an important cause of heart valve disease globally. Rheumatic heart disease is the most common etiology worldwide [1]. Though its prevalence has been steadily decreasing in developed countries, it is still frequent in low-income countries. The prevalence of degenerative mitral stenosis increases with age, and is a common finding in the elderly population in developed countries [2]. Both types of mitral stenosis are more frequent in females [3]. Less common causes of mitral stenosis include: congenital causes, systemic immune-mediated diseases, carcinoid syndrome, radiotherapy, and some drugs.

Echocardiography is the main imaging modality to diagnose and assess the severity and hemodynamic consequences of mitral stenosis [4,5] as well as valve morphology. Transthoracic echocardiography (TTE) is sufficient for the management of most patients. The focus of this review is the role of current two-dimensional (2D) and three-dimensional (3D) echocardiographic imaging for the evaluation of mitral stenosis.

## 2. Rheumatic Mitral Stenosis

### 2.1. Morphological Findings

**Figure 1:**
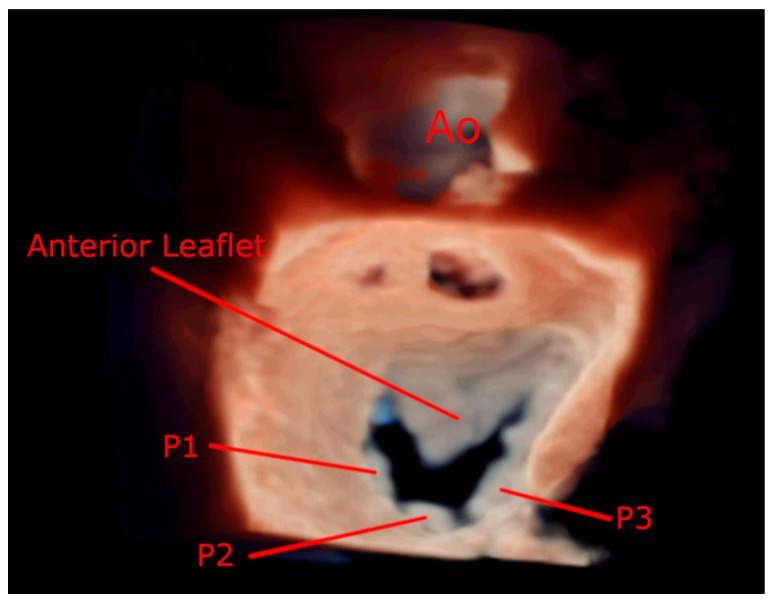
Zoom 3D image focused on the mitral valve from the mid-esophageal, four chamber view depicting mitral valve anatomy.

Knowledge of normal mitral valve anatomy is useful to appreciate the significance of disease-induced changes. The normal mitral valve annulus area is found to be on average 10 cm^2^ (rather than 4–6 cm^2^ as previously thought) [6]. The normal decrease in the annular area in systole of the mitral annulus is about 25%. The mitral valve comprises two leaflets, anterior and posterior, tendinous chords, and the papillary muscles. The anterior leaflet has a semicircular shape and is larger than the posterior leaflet: it comprises one-third of the annulus but two-thirds of the mitral valve orifice. It is divided arbitrarily into three segments, scallops, labelled A1, A2, and A3 from anterolateral to posteromedial. The posterior leaflet comprises the other two thirds of the annulus, and has indentations that define the three scallops; P1, P2, and P3 (Figure 1). Normal leaflet thickness is up to 4 mm [7].

Echocardiography provides key information regarding valvular morphology, subvalvular apparatus, leaflet thickness and mobility, and degree and location of calcifications. Echocardiographic findings in mitral stenosis are related to etiology. The main echocardiographic characteristic of rheumatic mitral stenosis are:

- Commissural fusion with valvular thickening, particularly at the free edge of the leaflets. This is usually best seen in the parasternal short-axis view. Later, thickening extends towards the base with further restriction of valve motion.
- Reduced leaflet mobility and restricted opening. The posterior leaflet is usually partially or completely immobile and the anterior leaflet shows a diastolic doming, producing the characteristic “hockey stick” configuration. This is usually best seen in the parasternal long-axis view.
- Affection of the subvalvular apparatus with thickened, fused, and/or shortened chords.
- Calcification of the leaflets and the subvalvular apparatus.

### 2.2. Evaluation of the Degree of Mitral Stenosis

#### 2.2.1. Mitral Valve Area

##### Planimetry

Direct planimetry of mitral valve area is the reference method to evaluate mitral stenosis severity [4]. 2D planimetry is performed in the parasternal short-axis view. It involves tracing the inner edge of the mitral valve orifice in mid diastole. The measurement has to be made at the leaflet tips, ensuring the narrowest valve orifice. In order to select an adequate alignment of the valve orifice plane, a careful scanning from base to apex should be performed. Zoomed images should be acquired and an excessive gain setting should be avoided as it may lead to underestimation of mitral valve area.

2D planimetry is a direct measurement. It is independent of load conditions, chamber stiffness and concomitant valve disease and reliably correlates with the size of the anatomic orifice [8]. On the contrary, an adequate acoustic window is needed, and severe calcification or distortion of the leaflets may lead to measurement errors. Inadequate alignment of the image plane is the main source of error. Due to these limitations, the severity of mitral stenosis should not be defined solely by this parameter, and an integrated approach is recommended.

##### Pressure Half-Time (PHT)

PHT refers to the time required for the peak mitral gradient to drop by one half. The more severe the stenosis, the slower the left ventricle will fill and the longer the time required for the transmitral gradient to decrease. PHT has been empirically correlated with mitral valve area with the Hatle formula: mitral valve area = 220/PHT. From the practical point of view, PHT is obtained by tracing the deceleration slope of the E wave. Thus, an accurate recording of the continuous wave Doppler diastolic mitral inflow should be obtained. In non-linear deceleration slopes, such as in cases of a bimodal shape of the E slope, a mid-diastolic line-drawing method is a reliable technique for measuring PHT [9]. Furthermore, in patients with atrial fibrillation, short cardiac cycles should be avoided, and several cardiac cycles should be averaged.

One advantage of this method is that it can be performed in cases where the acoustic window makes direct planimetry difficult. On the contrary, PHT is not dependent only on the mitral valve area, but also on the left atrium and left ventricle, and peak mitral gradient. Thus, in presence of a left ventricle restrictive filling pattern, low left atrium compliance, atrial septal defect or significant aortic regurgitation, the PHT is shortened and mitral valve area is overestimated.

##### Proximal Isovelocity Surface Area (PISA)

This method assumes that blood flowing towards an orifice accelerates, forming concentric hemispherical layers. These layers are called proximal isovelocity surface areas, and blood velocity is equal for each of them. These areas can be studied by color Doppler. Color baseline should be adjusted to better define a hemisphere.

Mitral valve area can be calculated by: mitral valve area = α/180 · V_aliasing_ ∙ πr^2^/peak V_mitral_, where r is the radius of the hemisphere and α is the opening angle of mitral leaflets relative to flow direction.

PISA method is rarely used in mitral stenosis, though it can be useful in the presence of significant mitral regurgitation.

##### Volumetric Method

This method assumes that filling volume of diastolic mitral flow is equal to aortic stroke volume. Stroke volume is calculated by multiplying the velocity time integral (pulsed wave Doppler) and left ventricular outflow tract area. Mitral flow can be calculated in the same manner. Thus, VTI_Mitral_ · Area_Mitral Valve_ = VTI_Aorta_ · Area_LVOT_. It is important to note that the velocity time integral of the mitral flow should be obtained at the level of the mitral valve orifice and not at the level of the mitral leaflet tips. Despite the appealing simplicity of this method, it is rarely used in clinical practice. It cannot be used in the presence of significant mitral or aortic regurgitation, and the number of required measurements increases the probability of error.

#### 2.2.2. Mitral Gradients

Mitral gradients play an important role in the assessment of mitral stenosis severity. Peak and mean mitral gradients are obtained by tracing the continuous wave Doppler across the mitral valve in the apical four chamber view using the simplified Bernoulli equation. Careful attention must be paid to beam alignment. Mean gradient is the most important hemodynamic parameter to assess mitral stenosis severity. Peak gradient is more dependent on left atrium and left ventricle compliance and loading conditions, and as such is more variable.

Transmitral gradients are highly dependent on heart rate. Changes in hear rate explain a substantial proportion of the variability. Therefore, the heart rate at which the gradients were measured should be reported. In patients with atrial fibrillation, mean gradient should be calculated as the average of five cycles with the least variation of R–R intervals and as close as possible to normal heart rate [10]. The transmitral gradient is also influenced by flow, and in the presence of mitral regurgitation, may lead to overestimation of the severity of mitral stenosis.

#### 2.2.3. Additional Parameters

##### Left Atrium

Significant mitral stenosis produces gradual left atrium enlargement. When assessing left atrium size, a measurement of the volume is recommended. Left atrial volume is obtained by tracing the endocardial border of the left atrium in both the apical four chamber and two chamber views by the application of the biplane method of discs (modified Simpson’s rule) [11]. The measurement should be made on the frame just before mitral valve opening. Careful attention must be paid to avoid foreshortening, and dedicated views focused on the left atrium are recommended.

##### Pulmonary Artery Systolic Pressure (PSP)

In the absence of an alternative cause, the presence of pulmonary hypertension is an indicator of the hemodynamic consequences of mitral stenosis, and suggests the presence of a significant degree of stenosis. Thus, PSP should be routinely estimated in patients with significant mitral stenosis. It is important to note however, that PSP resting values may be normal even in the presence of severe mitral stenosis.

Later in the course of the disease, a dilated and dysfunctional right ventricle and secondary tricuspid regurgitation may be found. Tricuspid regurgitation may also be a result of primary rheumatic involvement of the valve.

## 3. Degenerative Mitral Stenosis

In recent years, the epidemiological scenario of mitral stenosis has been changing in developed countries: the prevalence of rheumatic disease is decreasing, and the prevalence of degenerative mitral stenosis related to mitral annulus calcification is increasing. This is generally seen in the elderly and in patients with severe renal disease. It is regarded as an active and regulated molecular process of injury, lipid deposition, inflammation and bone formation [12].

There are distinct echocardiographic features that differentiate rheumatic and degenerative mitral stenosis. As opposed to rheumatic stenosis, the main lesion is annular calcification, predominantly along the posterior aspect, resulting in a reduced annular area. Rarely, it extends anteriorly, and severe forms may involve the leaflets. Commissural fusion is not present and leaflet tip motion is relatively preserved. These forms are most often mild or moderate.

The echocardiographic evaluation of degenerative mitral stenosis severity is difficult. As previously mentioned, rheumatic mitral stenosis severity can be assessed by planimetry, PHT, the volumetric method, the PISA method, and the mean transmitral gradient. However, these parameters lack validation in degenerative mitral stenosis.

Direct planimetry is challenging in these patients because of acoustic shadowing produced by calcium. Moreover, degenerative mitral stenosis is associated with advanced age, and diastolic dysfunction is not uncommon. PHT may lead to an overestimation of mitral valve area in conditions of elevated left ventricle filling pressure or abnormal left atria compliance [13]. The volumetric method has the same limitations in patients with rheumatic disease: the probability of error is high and it cannot be used in the presence of significant mitral or aortic regurgitation, which are common occurrences in this population. Finally, the PISA method can be challenging due to extensive calcification, and it has not been validated for this purpose.

Mean transmitral gradient is a useful parameter for evaluating degenerative mitral stenosis. In patients with mitral valve dysfunction related to annular calcification, mean transmitral gradient is associated with increased mortality after adjustment for age, sex, and risk factors related to mitral annular calcification [14,15]. It is important to note that when mitral annulus calcification is present, numerous confounders influence the transmitral gradient and render the correlation between mean gradient and mitral valve area less predictable [16].

## 4. Congenital Mitral Stenosis

Congenital mitral stenosis is a rare congenital heart malformation that comprises a heterogeneous group of lesions that usually present in association with other congenital diseases such as left heart underdevelopment, atrioventricular canal defects, left ventricular outflow tract obstruction, or Shone complex. Congenital mitral stenosis rarely occurs in isolation.

Shone complex is a rare congenital heart disease that comprises coarctation of the aorta, valvular and subvalvular aortic stenosis, and mitral stenosis either as a parachute mitral valve or as supravalvar mitral membrane (supramitral ring).

Parachute mitral valve is an anomaly in which all the tendinous chords of the mitral valve are attached to a single papillary muscle and is a common finding in the setting of congenital mitral valve stenosis.

## 5. Two-Dimensional Transthoracic Echocardiography Assessment

Two-dimensional transthoracic echocardiography is the first-line method used to evaluate mitral valve morphology and to diagnose and assess the severity and hemodynamic consequences of mitral stenosis. In order to perform a comprehensive assessment, multiple scanning planes should be explored:

### 5.1. Parasternal Long-Axis View

**Figure 2:**
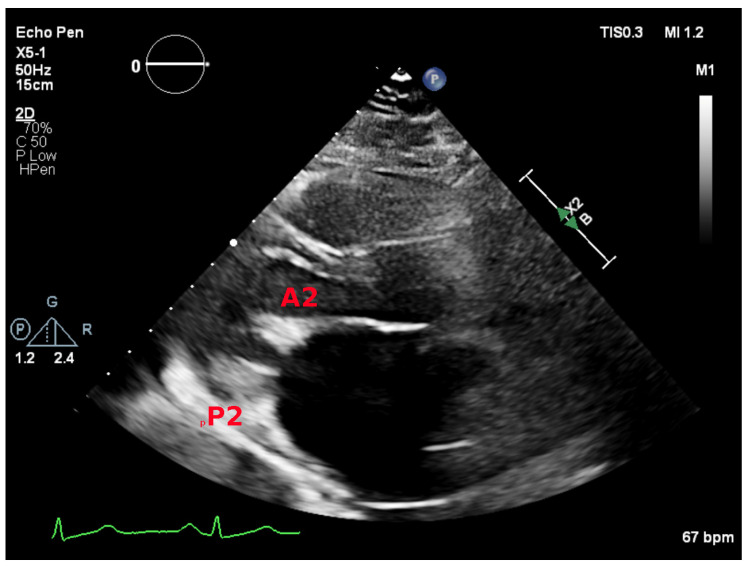
Parasternal long-axis view showing the A2 and P2 mitral scallops.

The parasternal long-axis view allows for visual assessment of mitral valve morphology and leaflet excursion. It demonstrates A2 and P2 scallops. Leaflet motion and thickness and the presence and extend of calcification should be reported. The characteristic doming of the anterior leaflet is better seen in this view (Figure 2). In mitral stenosis, color Doppler demonstrates diastolic turbulence across the mitral valve in this view.

### 5.2. Parasternal Short-Axis View

**Figure 3:**
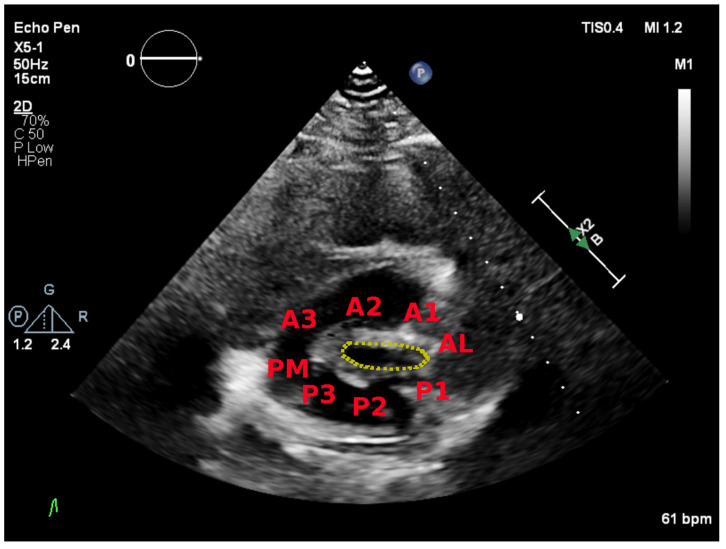
Parasternal short-axis view showing the posteromedial (PM) and anterolateral (AL) commissures and the mitral scallops.

The parasternal short-axis view allows for visual assessment of the leaflets and commissures. Commissural fusion is best seen in this view. The posteromedial (left) and anterolateral (right) commissures are visualized. Scallops one to three are seen from the anterolateral commissure to posteromedial commissure. This view is also used to perform planimetry of the mitral leaflet tips. Tilting from base to apex will demonstrate the narrowest valve orifice (Figure 3).

### 5.3. Apical Four Chamber View

**Figure 4:**
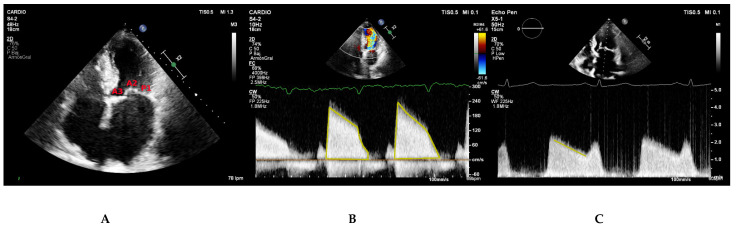
(**A**) Apical four chamber view depicting the P1, A2, and A3 mitral scallops. (**B**,**C**) Continuous wave Doppler parallel to the mitral inflow in apical four-chamber view. The Doppler profile (**B**) and the deceleration slope of the E wave (**C**) are traced to obtain the transmitral gradients and the PHT respectively.

The apical four chamber view typically demonstrates A3, A2, and P1 scallops from left to right (Figure 4A). It allows for visual assessment of the anatomy and mobility of both mitral leaflet. Continuous wave Doppler parallel to the mitral inflow in this view is used to measure transmitral gradients across the valve (Figure 4B). The densest portion of the curve should be used. Calculation of mitral valve area by PHT is also performed in this view (Figure 4C).

### 5.4. Classification of Mitral Stenosis Severity

Table 1 shows the classification of mitral stenosis severity according to mitral valve area and supportive findings.

### 5.5. Assessment of Feasibility of Percutaneous Mitral Commissurotomy

Suitability of mitral valve for percutaneous mitral commissurotomy is based on echocardiographic findings. Scoring systems have been developed to help with this task. The Wilkins score [17] (Table 2) is the most widely accepted criteria for patient selection.

Percutaneous mitral commissurotomy is contraindicated in patients with a mitral valve area >1.5cm^2^, left atrial thrombus, more than mild mitral regurgitation, severe calcification, absence of commissural fusion, severe concomitant aortic valve disease, severe combined tricuspid stenosis and tricuspid regurgitation requiring surgery or concomitant coronary artery disease requiring surgery [4].

## 6. Two-Dimensional Transesophageal Echocardiography Assessment

**Figure 5:**
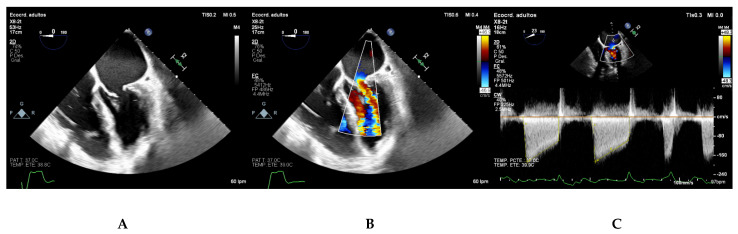
(**A**) Mid-esophageal four chamber view. (**B**) Color Doppler at the mitral valve level in a mid-esophageal four chamber view. (**C**) Continuous wave Doppler parallel to the mitral inflow in mid-esophageal four chamber view.

Transesophageal echocardiography should be performed to exclude left atrial thrombus before percutaneous mitral commissurotomy or after an embolic event [4]. Due to the absence of interference from the chest wall or the lung, transesophageal echocardiography offers a higher imaging resolution. Thus, it is also useful in patients with limited acoustic window or when clarification regarding valve morphology or suitability of mitral valve for percutaneous mitral commissurotomy (Figure 5).

## 7. Three-Dimensional Echocardiography Assessment

3D echocardiography represents a key innovation in cardiovascular imaging. The development of matrix-array transducers has allowed for real-time acquisition and online display of 3D images. Nowadays, 3D echocardiography is available on most ultrasound machines, and current transducers are able to acquire both 2D and 3D images. These technological advances have led to a progressive implementation of 3D echocardiography in routine clinical practice. Its main advantages are:

- It measures all three spatial dimensions. Therefore, it is not reliant on plane positioning and does not require geometric assumptions of cardiac structures.
- Images can be rotated and viewed from different perspectives. This allows for a better understanding of the relationship between structures and makes 3D images more intuitive.

On the contrary, limitations of 3D echocardiography include reduced spatial and temporal resolution compared to 2D echocardiography and suboptimal images in patients with arrhythmias.

First, 2D images should be used to locate the planes for imaging the mitral valve. Later, 3D imaging of the mitral valve can be performed using multiple 3D imaging modalities.

### 7.1. Simultaneous Biplane Imaging

Biplane mode allows for the use of a dual screen to display the mitral valve in two planes in real time. The second plane is usually orthogonal, though any rotation angle can be used. Color Doppler imaging can also be used together with biplane imaging. This mode is useful for a preliminary evaluation of the mitral valve. Scallops and segments of the mitral valve can be investigated by manipulating the lateral plane in different angle orientations in order to provide detailed information regarding mitral valve anatomy and the mechanism and etiology of mitral stenosis. In transthoracic echocardiography, biplane imaging can help ensure that planimetry takes place at the leaflet tips. An orthogonal plane can be obtained placing the cursor at the leaflet tips in the parasternal long-axis view. However, in order to avoid an oblique short-axis view, adequate alignment of the mitral orifice in the long-axis view is required.

### 7.2. Full Volume Imaging

Full volume imaging provides the largest sector and allows for an evaluation of the entire mitral valve apparatus. Real-time full volume acquisitions generally result in poor spatial and temporal resolution. Therefore, whenever possible, ECG gating acquisitions should be used. A full volume with gated acquisition is reconstructed by merging several individual volume slices gated to the ECG. This results in high temporal and spatial resolution. However, it can also be susceptible to artefacts due to arrhythmias and/or respiratory motions. ECG should be optimized to show a distinct R wave and the number of acquisition beats should be adjusted attending to the patient’s circumstances. Moreover, images should be obtained with breath-holding. Once acquired, the full volume dataset can be cropped, rotated, viewed in multiple perspectives, or decomposed into 2D cross-sections.

### 7.3. Real-Time 3D Imaging

Real-time 3D imaging permits a narrow sector and therefore higher spatial and temporal resolution. However, the sector is generally insufficient to visualize the entire mitral valve apparatus. The pyramidal volume should be acquired at a decreased depth and focused on the mitral valve. This allows for an initial 3D evaluation of the mitral valve.

### 7.4. Focused Wide-Sector (3D Zoom) Imaging

**Figure 6:**
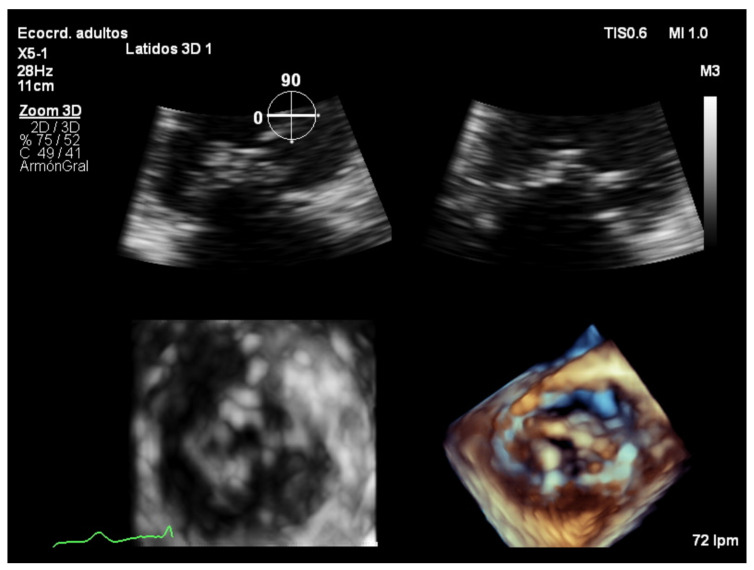
Zoom 3D focused on the mitral valve from the apical four chamber view.

**Figure 7:**
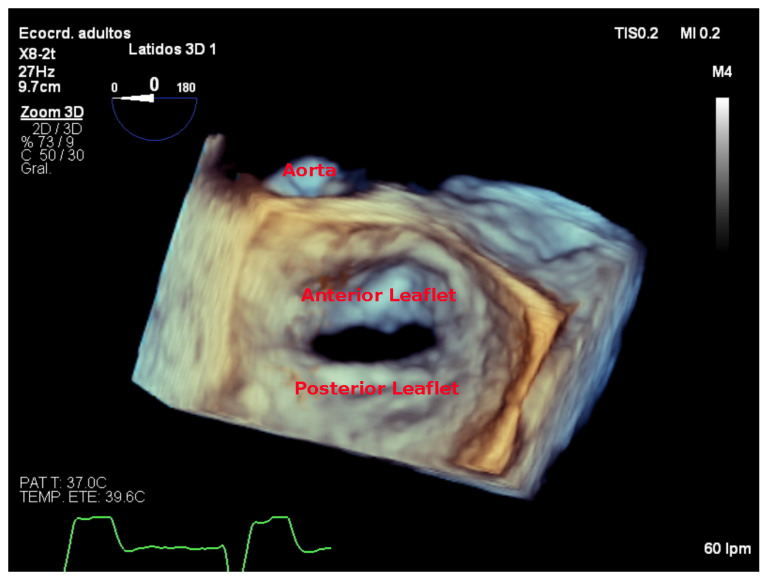
Zoom 3D focused on the mitral valve as view from the left atrium with the aorta placed superiorly (surgical view).

Focused wide-sector imaging allows for a 3D focused view of the mitral valve. In transthoracic echocardiography, 3D zoom of the mitral valve can be acquired from both the parasternal and the apical views (Figure 6). The anterior leaflet is adequately visualized in both views while the posterior leaflet is better seen from the parasternal view [18]. The mitral valve should be oriented with the aortic valve placed superiorly regardless of if the valve is oriented as viewed from the left atrium or the left ventricle (Figure 7) [19].

Three-dimensional echocardiography is superior in evaluating mitral stenosis severity, because direct visualization of the valve orifice in multiple planes ensures that the planimetry takes place at the level of the mitral leaflet tips, where the valve orifice is narrowest. Indeed, 3D planimetry has been reported as the most accurate method for mitral valve area evaluation [20,21], and it has become the reference method for mitral valve area quantification.

**Figure 8:**
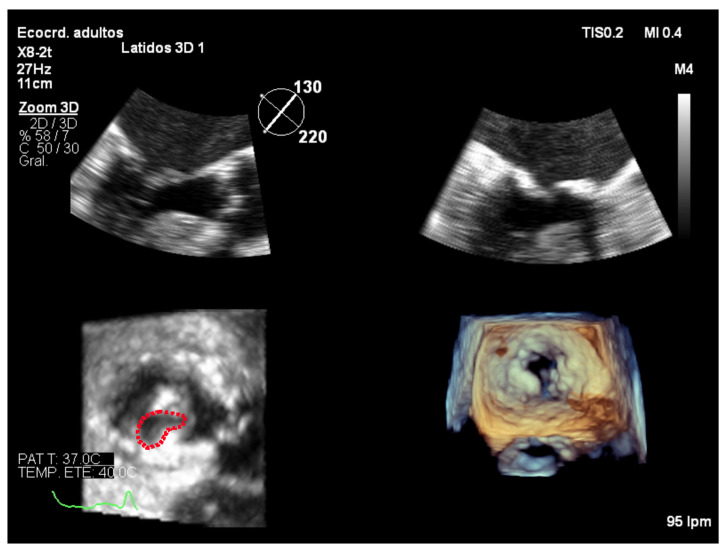
Zoom 3D focused on the mitral valve from the mid-esophageal four chamber view. Multiplanar reconstruction to perform 3D planimetry.

**Figure 9:**
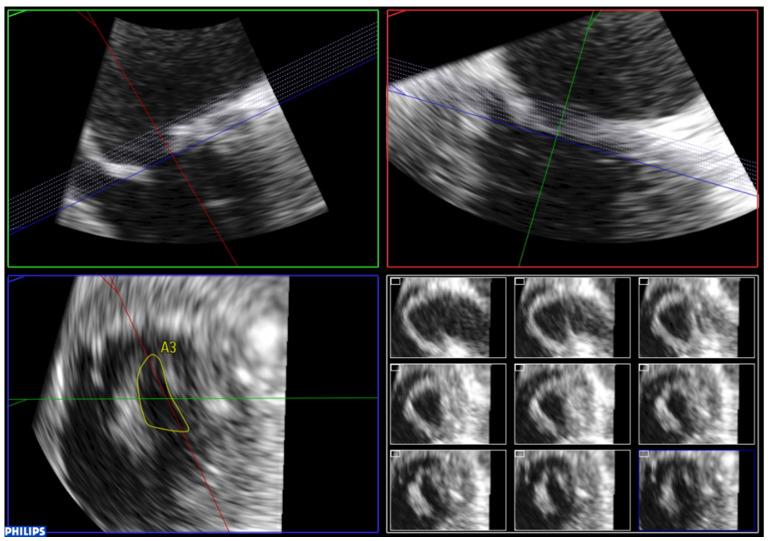
Analysis of zoom 3D dataset focused on the mitral valve obtained from the mid-esophageal four chamber view. Multiple short-axis slices are reconstructed to help identify the narrowest mitral valve orifice.

In order to perform 3D planimetry, the 3D zoom dataset should include the entire mitral annulus and the leaflet height. Later, the frame when leaflet excursion is greater should be identified and multiplane reconstruction should be used to achieve a parallel alignment with the narrowest portion of the mitral valve orifice. Finally, the mitral valve area is obtained tracing the inner edge of the orifice (Figure 8). Modern software permits multiple short-axis slices to be reconstructed to help identify the narrowest mitral valve orifice (Figure 9).

### 7.5. Assessment of Feasibility of Percutaneous Mitral Commissurotomy Using 3D Echocardiography

A scoring system based on 3D echocardiography, the Anwar score, has been developed for the evaluation of patients before percutaneous mitral commissurotomy [22] (Table 3).

## 8. Conclusions

Echocardiography is the main imaging modality to diagnose and assess the severity and hemodynamic consequences of mitral stenosis as well as valve morphology. Transthoracic echocardiography is sufficient for the management of most patients. Direct planimetry of the mitral valve area is the reference method used to evaluate mitral stenosis severity. The mean gradient is the most important hemodynamic parameter to assess mitral stenosis severity, and is a key parameter for evaluating degenerative mitral stenosis, where direct planimetry is difficult. Transesophageal echocardiography should be performed to exclude left atrial thrombus before percutaneous mitral commissurotomy or after an embolic event. Three-dimensional echocardiography is superior in evaluating mitral stenosis severity, because the direct visualization of the valve orifice in multiple planes ensures that the planimetry takes place at the level of the mitral leaflet tips, where the valve orifice is narrowest.

## Figures and Tables

**Table 1:** Classification of mitral stenosis severity at heart rates between 60 and 80 bpm and in sinus rhythm. Adapted from Baumgartnet et al. [10].

	Mild	Moderate	Severe
Specific Findings Mitral Valve Area (cm^2^)	>1.5	1.0–1.5	<1.0
Supportive Findings Mean Transmitral Gradient (mmHg) PSP (mmHg)	<5 <30	5–10 30–50	>10 >50

**Table 2:** Wilkins score. The total score is the sum of the items (ranging from 4 to 16). Adapted from Wilkins et al. [17].

Grade	Mobility	Thickening	Subvalvular Thickening	Calcification
1	Highly mobile valve with only leaflet tips restricted	Leaflets near normal in thickness (4–5 mm)	Minimal thickening just below the mitral leaflets	A single area of increased echo brightness
2	Leaflet mid and base portions have normal mobility	Mid leaflets normal, considerable thickening of margins (5–8 mm)	Thickening of chordal structures extending to one third of the chordal length	Scattered areas of brightness confined to leaflet margins
3	Valve continues to move forward in diastole, mainly from the base	Thickening extending through the entire leaflet (5–8 mm)	Thickening extended to distal third of the chords	Brightness extending into the mid portions of the leaflets
4	No or minimal forward movement of the leaflets in diastole	Considerable thickening of all leaflet tissue (>8–10 mm)	Extensive thickening and shortening of all chordal structures extending down to the papillary muscles	Extensive brightness through out much of the leaflet tissue

**Table 3:** Anwar score. The total score is the sum of the items (ranging from 4 to 16). Mild mitral valve involvement is defined as < 8 points, moderate involvement as 8 to 13 and severe MV involvement as > 14 points. Adapted from Anwar et al. [22].

	**Anterior Leaflet**			**Posterior Leaflet**		
Thickness (0–6) (0 = normal, 1 = thickened)	A1	A2	A3	P1	P2	P3
Mobility (0–6) (0 = normal, 1 = limited)	0–1	0–1	0–1	0–1	0–1	0–1
Calcification (0–10) (0 = no, 1–2 = calcified)	0–2	0–1	0–2	0–2	0–1	0–2
	**Subvalvular Apparatus**					
	**Proximal Third**		**Middle Third**		**Distal Third**	
Thickness (0–3) (0 = normal, 1 = thickened)	0–1		0–1		0–1	
Separation (0–6) (0 = normal, 1 = partial, 2 = no)	0–2		0–2		0–2	
